# Unveiling new genetic insights in rheumatoid arthritis for drug discovery through Taxonomy3 analysis

**DOI:** 10.1038/s41598-024-64970-0

**Published:** 2024-06-19

**Authors:** Justyna Kozlowska, Neil Humphryes-Kirilov, Anastasia Pavlovets, Martin Connolly, Zhana Kuncheva, Jonathan Horner, Ana Sousa Manso, Clare Murray, J. Craig Fox, Alun McCarthy

**Affiliations:** C4X Discovery Ltd, Manchester One, 53 Portland Street, Manchester, M1 3LD UK

**Keywords:** Medical genomics, Rheumatic diseases, Rheumatoid arthritis, Genetics research, Drug development, Computational biology and bioinformatics, Drug discovery, Target identification, Genetic association study, Genome-wide association studies

## Abstract

Genetic support for a drug target has been shown to increase the probability of success in drug development, with the potential to reduce attrition in the pharmaceutical industry alongside discovering novel therapeutic targets. It is therefore important to maximise the detection of genetic associations that affect disease susceptibility. Conventional statistical methods such as genome-wide association studies (GWAS) only identify some of the genetic contribution to disease, so novel analytical approaches are required to extract additional insights. C4X Discovery has developed Taxonomy3, a unique method for analysing genetic datasets based on mathematics that is novel in drug discovery. When applied to a previously published rheumatoid arthritis GWAS dataset, Taxonomy3 identified many additional novel genetic signals associated with this autoimmune disease. Follow-up studies using tool compounds support the utility of the method in identifying novel biology and tractable drug targets with genetic support for further investigation.

## Introduction

Attrition in drug development is a major issue for the pharmaceutical industry, particularly for complex diseases, with significant R&D resources spent on projects that do not deliver new medicines for patients. It is now widely accepted that genetic support for a therapeutic drug target leads to an increased probability of successful delivery to patients of a new medicine, with the potential to significantly reduce attrition^1,2^. These observations have directed the focus of drug discovery groups towards results from genetic studies of disease. As opposed to rare diseases, which are often caused by the dysfunction of a single gene, common diseases are complex traits influenced by the added contribution of many genetic variants. Conventional analyses of Genome-wide association studies (GWAS) have generated thousands of associations, initially based on the study of single-nucleotide polymorphisms (SNPs) arrays but increasingly using whole exome—or whole genome-sequence data^3^. A critically important observation is that > 90% of GWAS variants identified fall in non-coding regions of the genome and thus do not directly affect the coding sequence of a gene but rather accumulate in DNA regulatory elements and could for example disrupt binding sites for transcription factors likely regulating the expression levels of genes in a cell type-specific manner. Therefore, it is unclear which genes these variants regulate and in which cell types or physiological contexts this regulation occurs. This has hindered the translation of GWAS findings and insights into clinical interventions^4^.

Another key current limitation of using GWAS datasets to provide insights into relevant disease biology is that no analysis method can extract all the genetic information relevant to a disease embedded in the genetics, leading to the concept of ‘missing heritability.’ i.e. the genetic component of disease susceptibility that is calculated from family or twin studies, but cannot be fully accounted for by the known genetic associations detected.

Many explanations for this missing heritability have been proposed, including: the existence of many rare mutations with strong effect not captured by the standard GWAS analysis methodologies^5^, gene–gene interactions with strong effect (where the individual genes are not in themselves sufficiently strongly associated with the disease to be detected)^6–8^, or ‘omnigenic’ models proposing that many thousands of genes with small effect impact disease susceptibility such that even current meta-analyses are insufficiently powered to detect most of the signals^6–8^.

Based on twin and family studies, heritability of rheumatoid arthritis (RA) is estimated at ~ 60%, showing a significant impact of genetic variation on disease aetiology^9^. A number of RA GWASs have been published, with datasets that have included over 300,000 people^10–12^. The largest of these studies identified ~ 75 loci associated with diagnosis of RA (11 of which were novel) and while this is an important contribution to understanding the aetiology of the disease, the authors calculate that the total of all these findings accounts for 40–50% of the heritability thought to affect development of RA, leaving significant missing heritability still to be identified^12^.

Whatever the reasons for the missing heritability, it is clear that many different analytical approaches are required to maximise the extraction of genetic insights from disease datasets. To this end, C4X Discovery has implemented a unique method (called Taxonomy3) for analysing and visualising human genetic datasets for drug target identification. Taxonomy3 represents a significant departure from traditional GWAS. It employs a probabilistic mathematical framework to visualise high-dimensional genetic data. At its core, the method utilises individualized divergences and Log Bayes Factors (LBFs), an approach that transforms genotype data matrices into a data matrix and facilitates a nuanced comparison between genetic profiles. Distinct from conventional GWAS techniques that predominantly focus on frequency and association, Taxonomy3 allows to visualise the genetic architecture. In addition, Taxonomy3 scales principal components according to their corresponding eigenvalues, facilitating the interpretation of signal importance. For instance, the first principal component is scaled by the largest eigenvalue since it explains a component with the most variability. This allows for direct comparisons with subsequent scaled principal components, which diminish in size as they account for decreasing variability associated with case–control separation. That way it accentuates the evidential weight of genetic variants, thereby providing a refined visual aid, especially beneficial for complex traits like RA. This capability to handle complexities inherent in genetic data marks Taxonomy3 as a powerful tool for uncovering genetic targets. This methodological shift facilitates the identification of subtle genetic signals that might be overlooked in traditional analyses, making it particularly suitable for complex autoimmune diseases, where genetic contributions are diverse and often polygenic. The method has previously been applied to a range of complex disorders and clinical applications: Stevens-Johnson syndrome (SJS) and toxic epidermal necrolysis (TEN)^13^, drug-induced skin blistering disorders^14,15^, cutaneous adverse drug reactions^16^, carbamazepine hypersensitivity^17^, and phase 2a weight loss clinical trial data^18^. In this paper, we present details of application of Taxonomy3 to a rheumatoid arthritis case/control dataset, show the additional genetic insights generated by the method, and provide examples where the findings allow the potential initiation of drug discovery programmes based on drug targets identified through these new genetic findings.

## Methods

### Overview of Taxonomy3 method

Taxonomy3 is based on correlations of individualized divergences (named Log Bayes Factors, LBFs)^19^, and their Principal Component Analysis (PCA): the mathematical principle of the method has been described previously^20–22^. Briefly, for each genotype in a case/control group, the LBF is calculated, and for all subjects, each genotype is replaced by the calculated LBF for that genotype (for details see Supplementary Information). In this way, the initial genotype data matrix is transformed into a LBF matrix of the same dimension, representing the information contributed by each subject and SNP pertaining to the overall case/control distinction. LBFs in this context compare the likelihood of the data under two different hypotheses (for example, presence or absence of a genetic association). The log transformation is used for several reasons, including stabilising variance and making the scale of the data more manageable, which is especially important given the high dimensionality and complexity of GWAS data. This process is crucial for generating variance, both between and within groups, which is a key aspect of identifying significant genetic signals. A critical aspect of Taxonomy3 is the computation of individualized log divergences for each variable. This involves comparing the parameters of the marginal distribution of that variable under two different models. For instance, in a case–control study, these two models could represent the case group and the control group. Divergences are used in the literature to measure how different entities (e.g. SNPs) are from a representative benchmark such as the mean or median of the studied group. An ‘individualized divergence’ provides the researcher with a measure of how similar/different an individual entity is from that benchmark. Studying those individual divergences provides the researcher with an opportunity to explore not only coarse differences between groups but to pinpoint granular differences within the groups. Divergences may be defined in many different ways depending on the underlying scientific question and the explored statistical hypothesis^21,23^. Throughout this study ‘LBFs’ refer to the observed LBFs that are calculated directly from the data without adjusting for the frequency of variables. This approach is suitable when the aim is to capture the impact of rare genetic variants that are often underestimated in genetic analyses. LBFs are real numbers and have additive properties allowing the use of linear algebra tools. In Taxonomy3, LBFs are used to generate what is effectively a new empirical genetic model from the SNP data. This model is then used in further analysis steps such as eigen decomposition of correlations of LBFs (PCA), which is the preferred multivariate analysis method as it produces independent sets of correlated variables. To accurately determine how much each variable contributes to case/control distinction, we projected variable loadings on to the observed case/control direction in relevant dimensional space. Other datatypes (gene expression, clinical variables etc.) can also be transformed into LBFs, which in turn can be co-analysed alongside genotype LBFs^21^.

The principal component results are visualised using biplots to display the relative position of subjects and variables. From this, variables can be identified that are important for discriminating cases from controls. Statistical significance of the results is assessed by permutation of the case/control labels and re-analysis, which is performed 10 000 times. For each variable, p-values are obtained by comparing the true observed projected loading to the Gaussian mixture model (Mixmod software^24^) fitted to the distribution of the permuted projected loadings. The Family Wise Error Rate Šidák correction for multiple testing is used to define the genome-wide threshold for significant variables using the exact number of tests being carried out.

### Taxonomy3 analysis data management

#### Subjects and phenotype data

We analysed the rheumatoid arthritis (RA) dataset from the Welcome Trust Case Control Consortium (WTCCC), comprising RA cases (n = 1999) recruited from sites across the UK^11^. As controls, we studied UK National Blood Service (NBS) controls (n = 1480) from the same source. Both cases and controls were genotyped with the Affymetrix 500 K SNP chip (For more information on the cases and controls, see^11^). The WTCCC has limited phenotype data on the disease samples: disease status, age, sex and broad geographical region within Britain. The downloaded data were stored and analysed on the Amazon Web Services cloud computing facilities. To ensure security, the data were encrypted, stored behind a HIPAA-compliant firewall, and access to the data was restricted to a defined list of IP addresses.

#### Genotypes

The latest annotations file for the discontinued Affymetrix 500 K DNA chip was obtained from the manufacturer. We used genotypes derived using the Chiamo algorithm as in the original WTCCC analysis, discarding genotypes having a call probability lower than 90%^11^.

#### Univariate quality control

The case and control data were first subject to conventional data quality control. The objective of this procedure was to detect and remove potential biases that could undermine the analysis.

Sex: Checking for sex discrepancies is critical to Taxonomy3 as LBFs are not defined in the same way for X-linked SNPs in males and females. Sex was inferred for each patient from intensities of X-linked genotypes and X chromosome heterozygosity and checked against the reported sex.

Missing values: Subjects with a missing value rate above 5% were removed from the analysis.

Relatedness: The objective of this analysis was to detect and remove inter-related subjects/samples from subsequent analyses. We selected a total of 35,941 highly variable SNPs having a high Minor Allele Frequency (above 48%). We then determined the percentage of identical genotypes in all possible pairs of subjects.

Variables: Variables were excluded from the analysis for any of the following reasons:

1. Monomorphic in the whole population
2. Missing > 5% of values
3. SNPs departing from Hardy Weinberg Equilibrium in the control group (threshold *p* = 10^–8^).

In classical GWASs, SNPs having a low minor allele frequency (MAF) are usually removed due to power considerations. This is not necessary in Taxonomy3, as the method is not affected by rare variants.

#### HLA imputation

Human leukocyte (HLA) genotyping is not available in the WTCCC RA dataset, therefore imputation of antigen (HLA) genotypes from SNP genotype data was performed in-house using the HIBAG imputation method^25^ with pre-trained parameter estimates specific to Affymetrix500k for European ancestry.

#### Genetic ancestry

Taxonomy3’s focus on individual variations and the relative weighting of genetic variants can make it more sensitive to subtle population-level differences. This could potentially accentuate the differences arising from population structure and is accounted for in an additional QC step—HapMap co-analysis, which is similar to PCA-based techniques used in traditional GWAS. In Taxonomy3, however, genetic ancestries other than the main genetic ancestry that is being analysed are visualised and excluded and only the main homogenous population is progressed.

We carried out a co-analysis of the HapMap data with the RA (cases) and NBS (healthy controls) datasets, looking to define an ethnically homogenous cluster of subjects. The objective of the analysis was to position RA and NBS subjects within a Caucasian/non-Caucasian ethnic contrast and to define an ethnically homogenous subgroup of Caucasians. Cohorts included in HapMap co-analysis were as follows:

- Reference population: HapMap Caucasians (CEU)
- Contrast populations: HapMap Chinese (CHB), Japanese (JPT), Tuscan (TSI) and Africans (YRI)
- Unknown subjects: RA and NBS subjects

Appropriately matched case and control populations for the trait being studied were selected using probabilistically defined regions using the Mahalanobis distance around centres determined by k-means clusters for the reference population of interest. An appropriate α percentile was selected to retain the most subjects while minimising population heterogeneity. This approach was used to exclude subjects introducing bias due to non-disease related patient stratification and potentially confounding the case/control distinction.

### SNP-to-gene mapping

A custom SNP-to-gene mapping pipeline was used, which implemented both FUMA^26^ and variant to gene (V2G) from Open Targets Genetics^27^ to gather evidence. First, SNPs in high LD (r^2^ ≥ 0.6) with Taxonomy3 SNPs were extracted from the EUR population of 1000 Genomes Data Phase 3^28^. These SNPs were then mapped to genes (“Tax3 genes”) by genomic distance (< 40 kb from gene boundary + 1 kb promoter), predicted functional consequences (variant effect prediction—VEP^29^), significant cis e/pQTLs (Open Targets Genetics collection) in relevant tissues, and chromatin mapping (Open Targets Genetics collection). These measures were then weighted and used to calculate a prioritisation score TOPSIS from the MCDA R package^30^ based on a theoretical best and worst SNP-to-gene mapping. Standard evidence weights were based on Open Targets Genetics V2G scoring (functional prediction: 0.35, QTL evidence: 0.35, genomic distance: 0.2, chromatin proximity: 0.1). A TOPSIS score threshold of 0.4 was implemented to represent high quality mappings, whereby the SNP occurs within a gene or has a significant eQTL. This pipeline is available on GitHub (https://github.com/c4x-discovery/tax3-ra-publication).

### Assessment of novelty

To assess the novelty of the findings at the SNP and gene level, data curated by the Open Targets^31^ and Open Targets Genetics^27^ platforms were used. All reported GWAS results related to EFO_0000685 (rheumatoid arthritis) in European populations were downloaded using the Open Targets Genetics API (November 2021) and lead SNPs were subjected to our SNP-to-gene mapping pipeline. Open Targets Indirect Disease Association Scores for Tax3 genes were investigated for novelty at the gene level (Open Targets download version 06.21).

### Bioinformatic assessment of drug tractability

Target tractability details were downloaded from Open Targets^31^ (Open Targets download version 11.21) to identify potential modalities to drug genetic targets directly or via relevant biological interactions. Scores were allocated to terms to aid visualisation of druggable targets. (Approved Drug: 1, Advanced Clinical: 0.9, Phase 1 Clinical: 0.8, Structure with Ligand: 0.5, UniProt loc high conf: 0.5, Literature: 0.5, GO CC high conf: 0.45, High-Quality Ligand: 0.4, UniProt loc med conf: 0.4, UniProt Ubiquitination: 0.4, High-Quality Pocket: 0.3, UniProt SigP or TMHMM: 0.3, Database Ubiquitination: 0.3, Med-Quality Pocket: 0.2, GO CC med conf: 0.2, Half-life Data: 0.2, Druggable Family: 0.1, Human Protein Atlas loc: 0.1, Small Molecule Binder: 0.1).

### Network and pathway enrichment

Network expansion was conducted on the protein-coding Tax3 genes from SNP-to-gene mapping, using experimental protein–protein interaction data from IntAct^32^ (downloaded from Open Targets). A minimum IntAct score threshold of 0.5 was used to identify medium–high confidence known protein–protein interactions between Tax3 genes, and interactors were included that interacted with at least 2 Tax3 genes. This provided a network of potential interactors that may be occurring in relevant cells and tissues. Disease-relevant gene expression data was obtained from Genevestigator^33^. 511 mRNA-seq samples were retrieved from studies relevant to RA, including only human samples labelled as RA or healthy control. This relevant set included blood and synovial tissues and cell types. Gene expression-based clustering was performed on Tax3 genes + interactors using WGCNA^34^ and clusters were analysed in the context of the protein–protein interaction network. Network and pathway enrichment was performed using the anRichment R package^34^ using the built-in “GO” and “biosys” collections. Network visualisation and clustering was performed within R using igraph^35^ and visNetwork^36^.

### Cell assays

Preliminary validation of putative targets was performed using tool compounds in a cytokine release assay with peripheral blood mononuclear cells (PBMCs). Briefly, human PBMCs from healthy donors (n = 3) were prepared from buffy coats and resuspended in RPMI-1640 containing 10% FBS, 1% penicillin/streptomycin, 2 mM L-glutamine and 50 μM 2-Mercaptoethanol. 1 × 10^5^ cells were added per well to a 96-well flat-bottomed plate, and for stimulated wells, anti-CD3 (final concentration 0.25 µg/mL) was added to cells immediately prior to seeding to the plate. Compounds (BML-210, CTLA-4 Fc Chimera, C4X_17358 and Merimepodib) were solubilised in DMSO with the final vehicle concentration in wells of 0.1%. Treatments were added in triplicate to wells in a final volume of 100 µL per well. Cells were cultured for 72 h at 37 °C, 5% CO_2_. At the end of the culture period, cells were stained for viability using eBioscience Fixable Viability Dye eFluor 780, and supernatants were collected for subsequent assessment of cytokine production by multiplex using ThermoFisher custom ProcartaPlex kits.

## Results

### Univariate quality control

Subjects and patients: sex discrepancies were discovered in 19 subjects, 2 subjects had a missing value rate above 5%, and 5 subjects were found to be related: these subjects were removed from the analysis. A total of 26 subjects, predominantly RA patients, were removed due to univariate QC deviations.

Variables**:** The following steps were combined to establish the variables that were used in subsequent analyses (Table 1):

**Table 1:** Univariate QC procedures for SNPs (using the 90% genotype call probability threshold).

Filter	Number of variables*	Outcome
Monomorphic SNPs	15,858	Removed
> 5% missing value rate	17,485	Removed
SNPs departing from HWE in controls	3663	Removed

*There are some overlaps between these filters (e.g. a SNP may monomorphic and have > 5% missing data).

We obtained the latest annotations file from Affymetrix pertaining to their discontinued 500 K chip. Table S1 in the Supplementary Information shows descriptive statistics for merged NBS & RA datasets and Supplementary Table S2 shows the chromosomal location of available variables.

From an initial list of 500,306 variables, the final QC’d data for HapMap co-analysis included 480,785 variables.

### HapMap co-analysis

Chiamo generated genotype calls were used. A total of 480,785 SNPs shared by all datasets, non-monomorphic and having a total missing value rate lower than 5%, were analysed.

**Figure 1:**
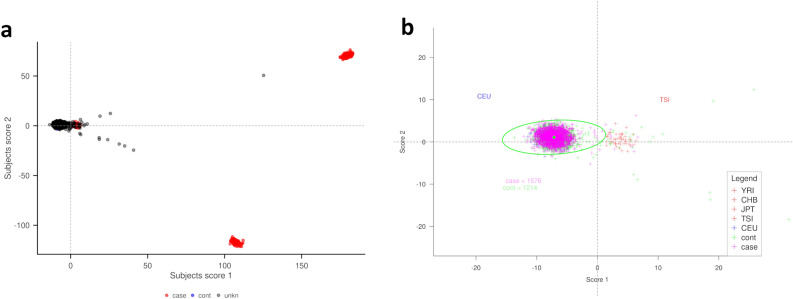
PCA score plot of Taxonomy3 co-analysis of HapMap, RA (case) and NBS (control) subjects. (**A**) Caucasian/non-Caucasian contrast with RA (case), NBS (control) and other genetic ancestries as unknowns—in correlation PCA. (**B**) PCA score plot of Taxonomy3 co-analysis of HapMap subjects. Patients within pink and green ellipses represent subjects associated with RA and NBS cohorts respectively, close to the CEU cohort. This is an expanded view of the region of interest.

Figure 1a shows the global Caucasian/non-Caucasian contrast. Subjects were somewhat dispersed, but the majority overlapped with the CEU cluster.

Figure 1b shows the genetic ancestry boundaries for cases and controls defined using a Mahalanobis distance from the Caucasian cluster centre. A percentile value of α = 0.2 was selected to define subjects in both cohorts using their respective ellipses.

The RA population in the WTCCC dataset was heavily skewed towards females. To reduce the chance of spurious associations cases and controls were matched with respect to sex by balancing the cohort to achieve an odds ratio of 1.

The output from the HapMap co-analysis gives a final population of 2005 subjects (Table 2) Subsequent Taxonomy3 analyses were restricted to these subjects.

**Table 2:** Final case/control cohorts.

	Cases	Controls
Pre univariate QC	1860*	1480
Post univariate QC	1834	1471
Post HapMap	1563	1212
Post HapMap sex balanced	793	1212

*1860 subjects available from the initial 1999 after applying manufacturer’s exclusion criteria.

### Taxonomy3 analysis results

**Figure 2:**
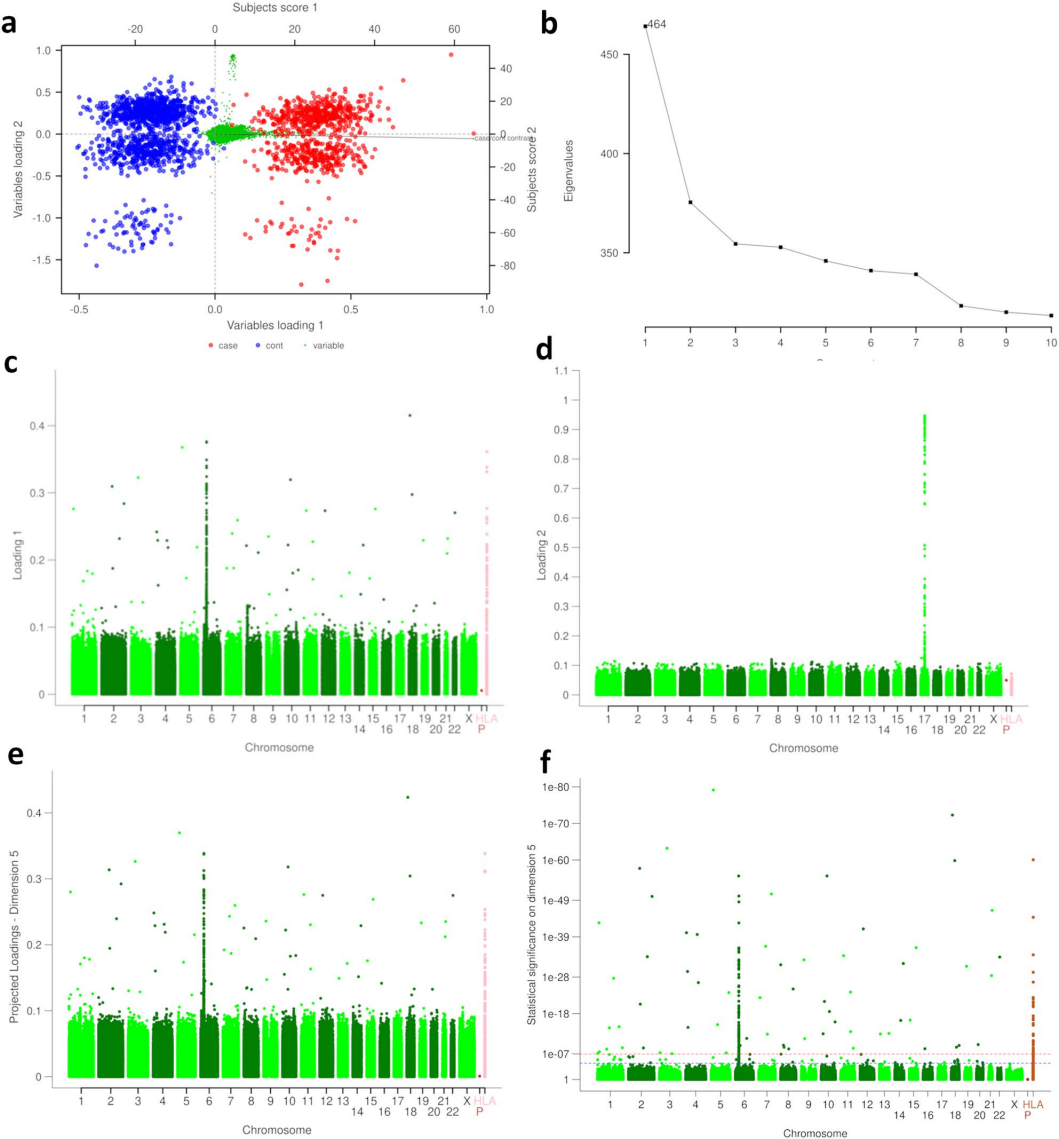
Taxonomy3 analysis plots. (**A**) Biplot of the first two PCA components, showing NBS controls (blue), RA cases (red) and SNPs variables (green). (**B**) PCA Scree plot, showing that most of the signal was retained by the first few principal components. (**C, D**) Manhattan plots for loadings 1 and 2. (**E**) Projected loadings plot showing dimension 5. (**F**) Manhattan plot based on 10,000 permutations of the case/control status representing projected loadings as p-values. The red and blue dotted lines represent the Šidák and Benjamini–Hochberg whole genome statistical thresholds, respectively. Plotted as HLA are imputed HLA variables and as *P*-data collection centres.

The primary output of Taxonomy3 analysis is shown in the biplot in Fig. 2. The cases and controls are well separated by the 1st principal component (PC1), and the case/control dummy variable, which is a categorical representation used to differentiate between case and control groups in the dataset. When we observe the alignment of this dummy variable in our PCA biplots, particularly against principal components, it indicates which components are most influential in distinguishing between cases and controls. In Fig. 2a that variable is almost horizontally aligned with the X-axis, showing that case/control separation is the biggest source of variation in the dataset and that it is captured almost exclusively in PC1 (Fig. 2a). This is confirmed by the Scree plot (Fig. 2b) which shows that most variation between case and controls is accounted for by PC1, with much smaller contributions from the other components. Therefore, variables projecting from the central origin along the case/control axis are relevant to discriminating cases and controls. This significant portion of explained variation underscores the effectiveness of Taxonomy3 in delineating between case and control groups. 2nd principal component (PC2) reveals a clustering pattern splitting both cases and controls into three distinct subgroups. Figure 2c and d show the loci driving the separation in PC1 (loading 1) and PC2 (loading 2), respectively. Signals found on PC2 are orthogonal to the case/control dummy variable and subsequently to the risk SNPs found on PC1, therefore have a very modest contribution to case–control separation. Figure 2e introduces a projected loadings plot with dimension 5. Projected loadings refer to the visualisation technique used in PCA to display the contribution of each variable to the principal components. Dimension 5 denotes the fifth principal component that has been extracted from the dataset. This component is part of the higher-dimensional space that PCA creates to capture the variance in the data pertinent to case/controls distinction. Here, dimension 5 captures most of the signal that distinguishes between the case and control groups and additional dimensions (dim6, dim7, etc.) do not contribute to this distinction (Supplementary Fig. 1).

**Figure 3:**
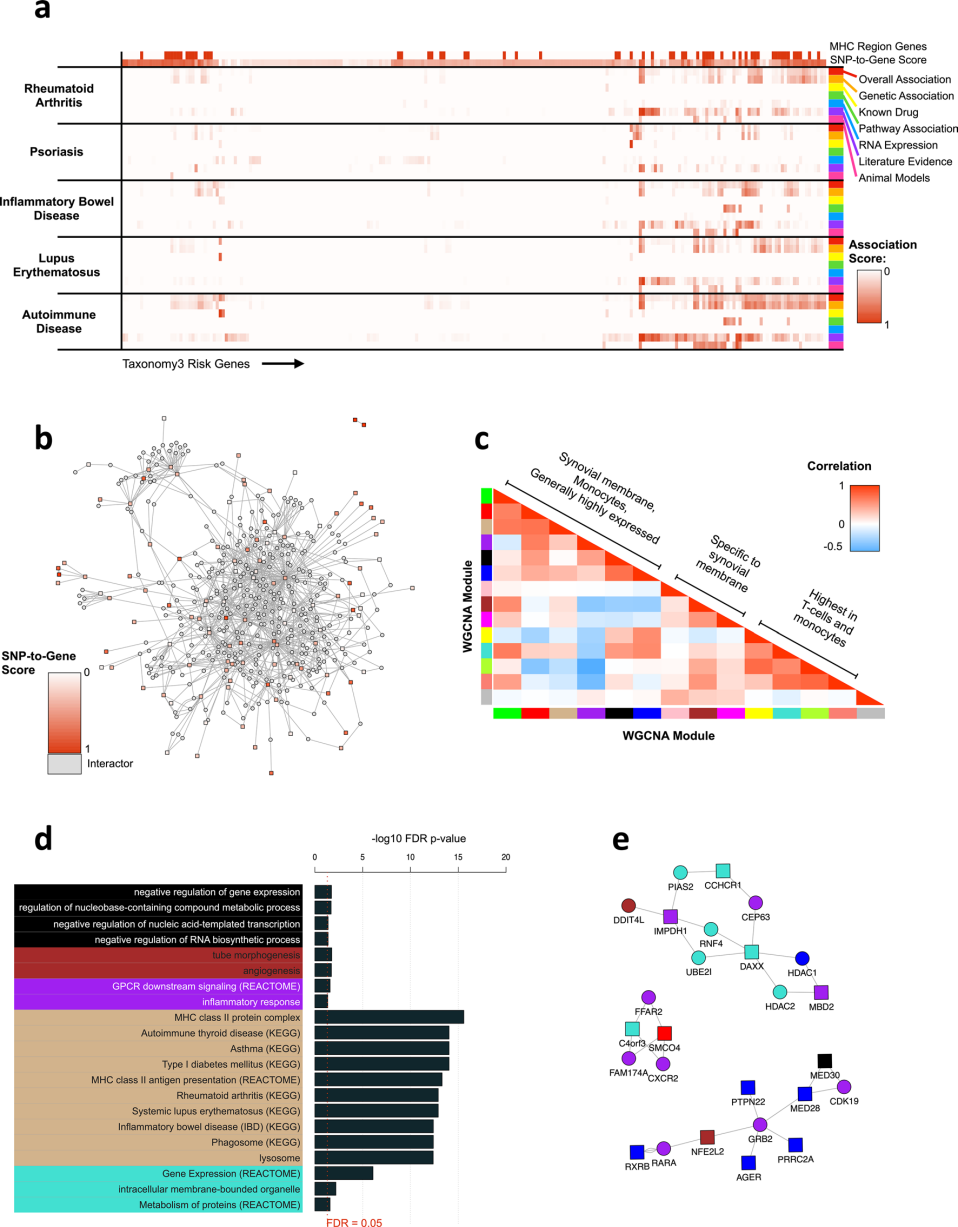
In-silico Exploration of Taxonomy3 Findings. (**A**) Known disease associations with genes mapped from Tax3 SNPs, using data from the Open Targets platform. (**B**) Network of protein–protein interactions between Tax3 genes (red/pink) and known interactors, using data from the IntAct database. (**C**) Broad clustering of WGCNA co-expression modules, showing enrichment across various tissues relevant to RA. (**D**) Significantly enriched terms for WGCNA co-expression modules. (**E**) Network expansion of purple module genes. Square nodes represent Tax3 genes and circular nodes represent interactors.

Permutation analysis was used to determine statistical significance of these findings. A total of 10,000 permutations of case/control labels and re-analysis were carried out. Permutation analysis plays a critical role in distinguishing true genetic associations from false positives. By randomly shuffling the case and control labels of our dataset and recalculating the association statistics for each SNP, we can create a null distribution of associations that would be expected by chance alone. By comparing our original association results to this null distribution, we can more accurately assess the likelihood that the observed associations are due to genuine biological signals rather than random fluctuations. This rigorous statistical method helps to reinforce the validity of our findings and is an essential step in the fine-mapping process to pinpoint potential causal variants. Based on the inspection of the Scree plot (Fig. 2b), 5 PCA components were retained. The Šidák genome wide significance threshold was used (alpha = 5%, *p* value = 1.13e−07). The resulting Manhattan plot is shown in Fig. 2f. Statistically significant loci were spread across the genome with a large LD block located on chromosome 6. Many of the loci on chromosome 6 are located within the major histocompatibility complex (MHC) region as indicated by HLA imputed variables (on the Manhattan plot Fig. 3e plotted as chromosome HLA).

### Interpretation of Taxonomy3 findings (SNP-to-gene)

Taxonomy3 genetic findings were mapped to genes using the parameters and pipeline described in Methods. The combined Taxonomy3 analysis revealed 173 SNPs that exceeded the Šidák significance threshold. As expected, and consistent with variants detected by conventional analysis, all of these SNPs except for one SNP (rs1800416) are located in non-coding regions of the genome. 109 significant SNPs mapped to the HLA region on chr6. The HLA region presents a challenge for SNP-to-gene mapping, as it is highly polymorphic, has a very high gene density and a low recombination rate resulting in a strong LD structure. Using the SNP-to-gene thresholds detailed in methods, a list of 233 genes was obtained (190 protein-coding genes).

### Novelty of Taxonomy3 findings

The novelty of Tax3 SNPs were assessed in two ways, by colocalising Taxonomy3 genetic signals with published GWAS results, and using gene-level disease association scores from Open Targets^31^ to explore known associations (genetic and others) between Tax3 genes and RA and related autoimmune disorders.

First, Tax3 SNPs were compared to significant GWAS results by matching exact rsIDs or matching rsIDs to SNPs in high LD (r^2^ ≥ 0.6 in 1 KG Ph3 EUR). Using this method, 2 loci within the MHC region were matched (rs6457620/HLA-DQB1 and rs9268557/HLA-DRA) and 1 locus on chr1 (rs6679677/PTPN22). To expand this genetic mapping, significant GWAS SNPs were mapped to genes using the same pipeline as Tax3 SNPs. This identified 6 additional loci outside the MHC region mapping to the same top genes, RTN4IP1 and SUPT3H on chr6, ACOXL on chr2, TTC34 on chr1, LYZL1 on chr10 and AMOTL1 on chr11.

For gene-level disease association, 3 related autoimmune disorders were selected (psoriasis, inflammatory bowel disease (IBD) and systemic lupus erythematosus) based on their potential overlap of disease aetiology with RA and the understanding of heritable elements for these diseases. The scores are hierarchically cumulative, so the *autoimmune disease* scores represent an accumulated score for all autoimmune disorders according to Open Targets^31^. Figure 3a displays a heatmap of these scores, excluding 86 Tax3 genes that have a value of 0 for all selected scores. SNP-to-gene evidence (TOPSIS) is also displayed, and MHC region genes are labelled. Rows and columns are hierarchically clustered using hclust from the fastcluster^37^ R package. This analysis revealed 3 additional weak known genetic associations with RA (chr2 rs4338920 LRP1B, chr13 rs17086772 SLC46A3 and chr4 rs17669915 LEF1). These weak associations are from GWAS for non-European populations, case-case comparative GWAS and similar disease GWAS. Other interesting genes without known genetic association with RA include 2 genes targeted by drugs in RA clinical trials (PDE5A: Dipyridamole, IMPDH1: Mizoribine), 1 additional gene targeted by drugs in clinical trials for other autoimmune diseases (ADRA1: Isoxsuprine for multiple sclerosis), 6 genes with known genetic association to autoimmune diseases but not RA (C1QTNF6, BCL2L11, KAZN, ETV3, MACROD2, PROK2, KCNE4), and 3 genes from pathways linked to IBD (PROK2, CYTH4, RAC2).

Taxonomy3 findings have been shown to support many existing genetic associations with RA, reveal unknown genetic associations with genes that are known to be associated with RA or related autoimmune disorders, and provide 150 novel, genetically associated RA targets for further validation and exploration.

### In-silico exploration of Taxonomy3 findings

Bioinformatics analysis was performed to interpret and prioritise the Tax3 genes. The 190 protein-coding genes underwent enrichment analysis as described in Methods. The top significantly enriched terms included Antigen processing and presentation (KEGG) (FDR *p* = 3.96e−03), MHC class II antigen presentation (REACTOME) (FDR *p* = 8.27e−03), and many KEGG terms for autoimmune and infectious diseases, including Staphylococcus aureus infection (FDR *p* = 3.96e−03), Autoimmune thyroid disease (FDR *p* = 8.27e−03), Systemic lupus erythematosus (FDR *p* = 1.69e−02), Asthma (FDR *p* = 8.27e−03), and Rheumatoid arthritis (*p* = 1.69e−02). These results were highly dominated with HLA genes, so a separate enrichment was performed excluding genes from the MHC region. The top terms for this analysis were transferase activity (*p* = 1.53e−03) and bone morphogenic protein (BMP) signalling pathway (*p* = 1.53e−03), the latter of which has been linked to autoimmune disease^38^.

Genetic association can only elucidate part of the mechanistic network for complex diseases, and our inclusive SNP-to-gene mapping pipeline will include false positives, so in order to contextualise our gene list and encourage dropout of false positives, a network expansion and clustering approach was undertaken. Experimentally validated protein–protein interactions (PPI) from IntAct^32^ were obtained between genes within the 190 protein-coding Tax3 genes, and also between interactor proteins and at least 2 Tax3 genes. The resulting PPI network included 114 Tax3 genes and 359 interactors. Network visualisation and clustering revealed some peripheral clusters, some clusters formed of pairs of Tax3 nodes with many common interactors, and a large central subnetwork of interconnected nodes (Fig. 3b). These interactions are mainly from in-vitro studies, so represent potential protein–protein interactions that could occur in a physiological context. To interpret this network and identify relevant clusters and subnetworks, 2 complementary approaches were undertaken, PPI network community identification using network clustering methods within igraph^35^, and network coexpression clustering using WGCNA^34^.

PPI subnetworks were identified using the cluster walktrap method from the igraph R package^35^. This identified 20 clusters with at least 5 members. For coexpression clustering, relevant gene expression data was downloaded from Genevestigator^33^. 511 patient samples from RA studies were selected, representing multiple disease-relevant tissue types, and 233 Tax3 genes + 349 interactors underwent network coexpression clustering using WGCNA^34^. This identified 14 clusters, 13 of which had distinct eigengene expression profiles in RA-relevant tissues. Correlation analysis of cluster eigengenes revealed 3 overall cluster groups (Fig. 3c) representing targets that were 1: widely expressed but highest in synovial membrane and monocytes, and low in fibroblast synoviocytes, 2: synovial membrane-specific, 3: highest expression in T-cells and monocytes, showing that Tax3 genes are enriched for genes expressed in highly disease-relevant cell types. 5 of these coexpression clusters had significantly enriched pathway terms, using a combined collection of GO, KEGG and REACTOME terms (Fig. 3d). The tan cluster was highly enriched for MHC class II genes, and many diseases for which MHC-II factors are associated. The purple cluster was enriched for inflammatory response genes (FDR *p* = 4.53e−02). This cluster did not feature any direct protein–protein interactions, but expanding this cluster for direct interactors displayed multiple purple interactor genes that may provide additional druggable targets to perturb the RA-specific inflammatory network identified through Tax3 genetic associations (Fig. 3e). Genes mapped from the MHC region represent distinct subnetworks of this analysis, as illustrated in Supplementary Fig. S2). This analysis provides potential drug targets that could be taken forward into a variety of assays to explore and validate their role in disease aetiology.

Small-molecule druggability (tractability) assessment was also conducted on network nodes using data from the Open Targets platform^31^ as described in the Methods. 3 additional interactor genes were identified that are reported to be targeted by drugs in clinical trials for autoimmune diseases (FGFR3: Masitinib for RA, KCNA3: Dalfampridine for multiple sclerosis, PSMB5: Bortezomib and Ixazomib for autoimmune thrombocytopenic purpura) In order to progress the analysis and validate potential therapeutic targets, genes with available tool compounds were selected that may impact and disrupt the disease network.

### In vitro validation of Taxonomy3 findings

In an attempt to validate the novel genetic associations from Taxonomy3 analysis in a preliminary cellular context, we identified those putative RA targets which were entirely novel, and those established RA targets without a previous genetic link that have been previously found to be expressed in leukocytes. Publicly available tool compounds were available for MEF2B and IMPDH1, and an internal compound (C4X_17358) was synthesized as an inhibitor of the potassium channel Kv1.3 (IC_50_ 2 nM determined via SyncroPatch, data not shown), of which the Tax3 gene KCNE4 is a functional co-factor (Table 3). These compounds were tested in the PBMC validation assay as described in the Methods. The study was designed to investigate potential effects of the tool compounds on immune cells within the PBMC population and so, anti-CD3 was used as the stimulus such that the co-stimulatory signal would be provided by antigen presenting cells within the culture rather than bypassing this by addition of anti-CD28. Anti-CD3 stimulation resulted in a robust increase in T-cell proliferation (Supplementary Fig. S3). CTLA-4 Fc Chimera was included in the study as a positive control^39,40^. Whilst it is not certain that the putative genetic variants modulate the mapped genes in PBMCs specifically, using tool compounds to investigate potential modulation of inflammatory pathways in these cells would provide justification to investigate this further.

**Table 3:** Taxonomy3 targets to be tested in PBMC validation assay.

Target	Tool compound	Genetic link to RA	Established mechanism in RA
MEF2B	BML-210^41^	No	No
Kv1.3 (KCNE4)	C4X_17358	No	Yes^42^
IMPDH1	Merimepodib^43^	No	Yes^44^

**Figure 4:**
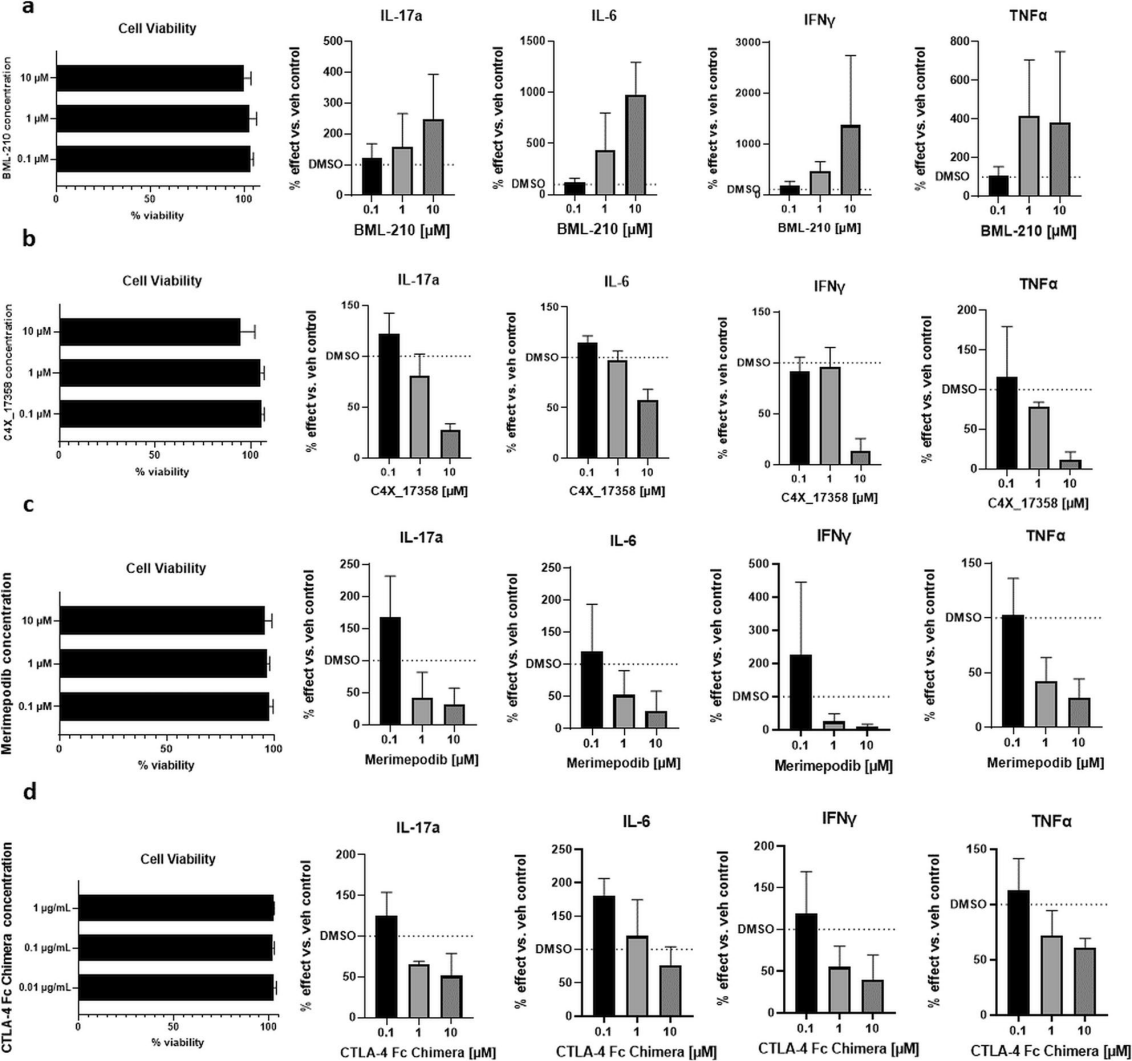
PBMC assay validation of Taxonomy3 targets. PBMCs were cultured, stimulated with anti-CD3 and treated with tool compounds as listed for 72 h. (**A**) BML-210 treatment. (**B**) C4X_17358 treatment. (**C**) Merimepodib treatment. (**D**) CTLA-4 Fc Chimera treatment. Viability of PBMCs was measured via eBioscience Fixable Viability Dye eFluor 780. Viability is represented as a percent of the viability of vehicle control cells. Levels of secreted IL-17a, IL-6, IFNγ and TNFα were determined via multiplex assays. Levels of cytokines are represented as a percent of cytokine secreted compared to vehicle control cells. PBMC assays were performed in technical triplicates, and an average of the three values taken to represent one biological replicate; three biological replicates were performed (three different donors).

Following 72 h of treatment, no compound was found to reduce cell viability below 75% (Fig. 4). Pro-inflammatory cytokine measurements were carried out for (interleukins) IL-17a, IL-6, interferon gamma (IFNγ) and tumour necrosis factor alpha (TNFα) (Fig. 4a–d) in cells stimulated with anti-CD3. BML-210 treatment resulted in a trend to further increase inflammatory cytokine release with increasing compound concentration (Fig. 4a). Comparatively, treatment with C4X_17358, merimepodib or CTLA-4 Fc Chimera resulted in a trend toward a decrease in pro-inflammatory cytokines with increasing compound concentration (Fig. 4b–d).

## Discussion

To date, divergences have been used in assessing diagnostic kit performance^45^, and more recently they have been used in Bayesian Analysis of Gene Essentiality (BAGEL) methodology to analyse pooled CRISPR studies^46^. Taxonomy3 is the first application of which we are aware that applies individualised LBFs to human genetic data linked to a binary outcome to be used in drug discovery. Replacing individual genotypes with the corresponding LBF and analysing the LBF matrix using PCA generates a number of outputs:

- Variables of interest relevant for case/control discrimination;
- Heterogeneity in the case/control populations, allowing for sub-groups within the populations to be identified;
- Variables of interest relevant for sub-group separation.

As this is a different approach to exploring genetic data, it is not surprising that the outputs are not identical to the output of the conventional GWAS analysis. In the original analysis of this RA case/control dataset^11^, two peaks were seen, one on chromosome 6 in the MHC region, and one on chromosome 1. The Manhattan plot from our Taxonomy3 analysis (Fig. 2a,e) shows that we have replicated these two findings and also greatly increased the number of significant associations. Unlike traditional GWAS, which primarily seeks to estimate parameters (like odds ratios) associated with SNP-trait linkages, Taxonomy3 places emphasis on deriving conclusions based on the data and available evidence. This shift in focus allows Taxonomy3 to provide insights into the distinctions between groups and the structure of individual observations within these groups. Comparison of our findings with the data currently available on Open Targets shows that while some of our additional findings have been observed in other, subsequent larger studies or meta-analyses (which provides additional validation for our methodology), there are other findings that provide truly novel insights.

Bioinformatic analysis of the Tax3 genes showed significant clustering in immune-related pathways (Fig. 3b), providing good validation for the newly identified genes. Likewise, co-expression clustering analysis showed that changes of expression of the genes were enriched in T-cells, synovial tissues and monocytes—highly disease-relevant cell types (Fig. 3c).

Suitable tool compounds were only available for a few of the Tax3 genes—Kv1.3 (KCNE4), IMPDH1 and MEF2B: two of these are of particular note:

KCNE4: Association with this gene was identified through Taxonomy3 analysis. This gene has not previously been associated with RA, and codes for an accessory protein modulating the activity of Kv1.3^47,48^. This K^+^ channel is important for the functioning of T_EM_ cells, which have a key role in maintaining the autoimmune drive in RA^49–51^. For this reason, inhibiting this channel has been proposed as a possible target for various inflammatory diseases^52–54^ including RA. We consider that the addition of genetic support for the pathway from our Taxonomy3 analysis significantly increases the likelihood of successful clinical development for inhibitors of Kv1.3.

MEF2B: This gene mapped from a SNP identified in Taxonomy3 analysis. The gene has not been associated with RA in published GWAS, and there is limited data to implicate the gene in RA. Examination of the tool compound BML-210, that blocks the interaction of MEF2 with histone deacetylase (HDAC), in in vitro models showed consistent effects of inhibition of MEF2B on immune cell function. This result underlines the ability of Taxonomy3 analysis to generate novel genetic insights, adding significantly to our knowledge of disease aetiology, and flagging novel drug targets with genetic support.

Association with NR4A3 was also identified in Taxonomy3 analysis passing the Benjamini–Hochberg genome wide significance threshold (false discovery rate correction). This nuclear receptor is known to impact expression of FoxP3, the key gene required for regulatory T-cell (Treg) formation^55–57^. A number of publications have demonstrated a dysfunction of Treg cells in RA patients^58–60^, and therapeutic modulation of Tregs for RA has been proposed^61^. Unfortunately, no suitable tool compounds were available to probe the functioning of this gene in the PBMC model.

There are a number of limitations to this study. Firstly, we have not been able to analyse other datasets (potentially from other genetic ancestries) to examine the translation of our findings to other populations. Secondly, as with all genetic studies, the inherent uncertainties of SNP-to-gene mapping means that the genes described here may not be the true genes involved in the genetic susceptibility detected by Taxonomy3. However, we have maximised the chance of including the causal gene by using a combination of positional and functional mapping in our SNP-to-gene process and downstream triaging to identify the genes with a high probability of impacting disease aetiology. Furthermore, as non-coding variants are thought to regulate genes in a cell type specific manner, it is possible that some or all of the subset of Tax3 genes we examined in the PMBC assay (MEF2B, Kv1.3 (KCNE4), IMPDH1) are in fact regulated by the variants detected, in a different cell type (e.g. fibroblast synoviocytes). Once confidence in a SNP-gene mapping is achieved, along with demonstrating clear impact of the target in a disease relevant context (conduct of additional studies extending beyond the remit of this publication) validation of the genetics will need to be confirmed e.g. show differential regulation of the gene in question depending on which allele is present. As the genotypes for the PBMC donors were not available this could not be examined alongside the tool compound examination. Extension of the studies to investigate the role of the genetic targets in PBMC from donors with RA, as well as identification of the cell types involved in the response for the individual targets, would also provide a greater level of support for the relevance of the targets in the disease setting.

In this paper, we describe a unique method of analysing genetic datasets. The results of our Taxonomy3 analyses present an opportunity for initiation of drug discovery programmes for treating RA, the identified novel targets (e.g. MEF2B) benefitting from the increased chances of success due to their genetic association with the disease, subject to further experimental validation. The method can be applied to all datasets with well-defined dichotomous cohorts e.g. case/control, mild/severe, responders/non-responders, and has the added benefit that other data types (e.g. mRNA expression, clinical data, longitudinal phenotypes) can all be converted into LBFs and co-analysed with genetic data^21^. The method can identify novel genes or pathways of interest for a disease (or other phenotype of interest) leading to innovative drug discovery programmes with the added confidence of genetic support.

### Supplementary Information

Supplementary Information.

## Data Availability

The data that support the findings of this study are available from Wellcome Trust Case Control Consortium but restrictions apply to the availability of these data, which were used under license for the current study, and so are not publicly available. Data are however available from the authors upon reasonable request and with permission of Wellcome Trust Case Control Consortium. A full list of the investigators who contributed to the generation of the data is available from www.wtccc.org.uk. Funding for that project was provided by the Wellcome Trust under award 076113. Accession numbers for data used in this study: EGAD00000000007 - WTCCC1 project Rheumatoid arthritis (RA) samples, EGAD00010000250 - NBS control samples.

## Code availability

The mathematical foundation of Taxonomy3 and its algorithms have been published previously^19–22^. The code used to run permutation analysis and bioinformatics analysis makes use of open-source software packages and is available at https://github.com/c4x-discovery/tax3-ra-publication. All analyses were performed on a cluster of Linux machines (Amazon Web Services).
